# A Mechanical Stimulation Glove to Induce Hebbian Plasticity at the Fingertip

**DOI:** 10.3389/fnhum.2020.00177

**Published:** 2020-05-25

**Authors:** Fabian Timm, Esther Kuehn

**Affiliations:** ^1^German Center for Neurodegenerative Diseases (DZNE), Magdeburg, Germany; ^2^Institute for Cognitive Neurology and Dementia Research (IKND), Otto-von-Guericke University, Magdeburg, Germany; ^3^Center for Behavioral Brain Sciences (CBBS) Magdeburg, Magdeburg, Germany

**Keywords:** tactile plasticity, skin stimulation, learning, rehabilitation, stimulation glove, tactile coactivation

## Abstract

Repetitive sensory stimulation of the fingertip induces Hebbian plasticity in the sensorimotor cortex that benefits the tactile and motor behavior of the hand in healthy younger adults, older adults, and patients. To use this method outside the laboratory, robust and portable stimulation systems are needed that allow prolonged stimulation phases over several hours without compromising on signal intensity or personal mobility. Here, we introduce two stimulation gloves that apply finger- and frequency-specific mechanical stimulation to individual fingertips over prolonged periods. The stimulators are built into commercially available cotton gloves and apply stimulation either via loudspeaker membranes or via linear resonant actuators (LRAs). We tested the efficiency of both gloves to induce Hebbian plasticity in younger adults by using two established measures of tactile performance, the grating orientation task (GOT), and the two-point discrimination task (2PDT). Both tests were performed before and after 3 h of sensory finger stimulation using one of either glove system. As a control condition, a non-stimulated finger was tested in both tasks before and after stimulation. The results show no significant effect of sensory stimulation on GOT thresholds, but a significant decrease in the 2PDT thresholds after compared to before the training at the stimulated finger only. The loudspeaker membrane improved performance in the 2PDT in 10/16 participants, whereas the LRA improved performance in the 2PDT in 13/16 participants. Stimulation gloves with built-in modules may be used in future larger-scale cohort studies on sensorimotor plasticity, rehabilitation, and learning.

## Introduction

During repetitive somatosensory stimulation, cortical areas that represent the site of sensory stimulation at the skin surface are targeted to induce neuroplastic processes. In such protocols, sensory stimulation is applied to a confined area of skin, such as the fingertip, for prolonged periods, which induces changes at the cortical level, such as the enlargement of finger representations in the primary and secondary somatosensory cortex (Pleger et al., 2003), reduced paired-pulse ratios (Höffken et al., 2007), and improvements in tactile, haptic, proprioceptive, and sensorimotor behavior (Dinse et al., 2006; Kalisch et al., 2008; Ragert et al., 2008). Repetitive somatosensory stimulation is assumed to induce long-term potentiation (LTP) via the intermittent high-frequency stimulation of one skin site that mediates N-methyl-D-aspartate-receptor (NMDA)-dependent Hebbian plasticity (Dinse et al., 2003). The advantage of repetitive somatosensory stimulation protocols compared to other available techniques is that the learning occurs in response to simple (passive) exposure to sensory stimulation but without the need for active training or task engagement (Beste and Dinse, 2013).

Therefore, somatosensory stimulation protocols have attracted interest in clinical settings, for example, to regain sensorimotor function after neuronal damage (Conforto et al., 2002, 2010; Wu et al., 2006; Kattenstroth et al., 2012). Conforto et al. (2010), for example, applied median nerve stimulation to the wrist of the paretic arm of stroke patients, in parallel to rehabilitative treatment, and reported dose-dependent beneficial effects of the stimulation on motor functions. In a recent study, Kattenstroth et al. (2018) used a novel stimulation glove to apply repeated electrical stimulation to the fingertips of stroke patients 3–4 weeks post-ictus. The authors reported significant enhancements in the tactile and motor behavior of stroke patients who underwent the stimulation protocol compared to those who did not (Kattenstroth et al., 2018). Stimulation gloves are therefore one potential method to apply repetitive somatosensory stimulation to patients in a clinical setting or to involve larger cohorts where patients apply the stimulation at their homes.

In previously introduced glove systems, electrical stimulators are used to provide repetitive somatosensory stimulation to the fingertips. Electrical stimulators, however, are vulnerable to humidity and heat, and require constant supervision and quality-checks due to safety reasons. Also, the effect of electrical stimulation on cortical activation is still debated, because it sometimes induces deactivations in the sensorimotor cortex (Klingner et al., 2011), and it represents an artificial rather than natural stimulus. The electrical protocol also uses high stimulation amplitudes that are usually set at the highest values that the patient can tolerate for an extended period (Kattenstroth et al., 2018), which introduces another risk factor for the wide applicability of this system in clinical settings, in rehabilitation, or at patients’ homes.

Here, we explore the effectiveness of a technology that is based on mechanical rather than electrical stimulation. Previous scientific protocols have applied repetitive mechanical stimulation to the fingertip to induce learning (e.g., Pleger et al., 2003; Kuehn et al., 2017). Mechanical stimulation is usually applied via a small loudspeaker membrane (diameter ~14.8 mm) that is fixated on the fingertip via adhesive tape to convey weak tactile stimuli to a small area of skin. Whereas this solution is suitable for laboratory-based studies, it is challenging to use for clinical settings or at patients’ homes due to the fragility of the thin loudspeaker membrane against external damage. The loudspeaker membrane often breaks after repeated testing and needs to be replaced frequently. Therefore, we searched for alternative solutions. The goal was to find a mechanical stimulation module: (i) that can be built into commercially available glove systems; (ii) that can be operated via battery or power bank and is therefore portable; (iii) that is robust against use-dependent damage; and (iv) that can apply different stimulation protocols to the fingertips. Here, we present one possible solution, which is a mechanical stimulation module based on linear resonant actuators (LRAs). LRAs apply mechanical stimulation via the vibration of a small metal disk that can be adjusted in frequency and amplitude. The system is thin yet more stable than loudspeakers due to the use of metal disks rather than thin membranes for inducing vibration. LRAs are lightweight and can be built into commercially available glove systems. The present study aimed to compare a glove system that has built-in LRA modules to a glove system that has built-in loudspeakers in their effectiveness to induce tactile learning at the fingertip. If both are effective, the LRA-glove may be preferred for future clinical applications due to the higher robustness of LRAs compared to loudspeakers, and due to the higher safety of LRAs compared to electrical stimulators, as outlined above.

An established protocol was used for stimulation, where for 3 h, repetitive somatosensory stimulation was provided to the fingertip. The average stimulation frequency was 1 Hz (varying between 0.3 Hz and 100 Hz), which stimulates different tactile receptor types in the skin, in particular Meissner corpuscles (response peak around 50 Hz) and Merkel nerve endings (response peak at around 5–10 Hz). The effectiveness of both glove systems to improve tactile spatial discrimination thresholds was assessed using two standard tests for spatial tactile acuity, the two-point discrimination task (2PDT), and the grating orientation task (GOT). 2PDT-thresholds and GOT-thresholds do not correlate and measure different aspects of tactile spatial acuity (Bruns et al., 2014). Both glove systems were also evaluated concerning their comfort and suitability to use during everyday tasks using questionnaires.

## Materials and Methods

### Participants

We tested *N* = 32 right-handed, healthy younger adults between 18 and 35 years (*n* = 22 females) who had no deficits in sensorimotor processing at the hand or any other body area. For *n* = 5 participants, no GOT threshold could be estimated due to low performance in this test; *n* = 4 additional participants were invited for replacement. All participants gave written informed consent and were tested following the Ethics guidelines of the Otto-von-Guericke University Magdeburg.

### Stimulation Gloves: Loudspeaker-Glove and LRA-Glove

**Figure 1:**
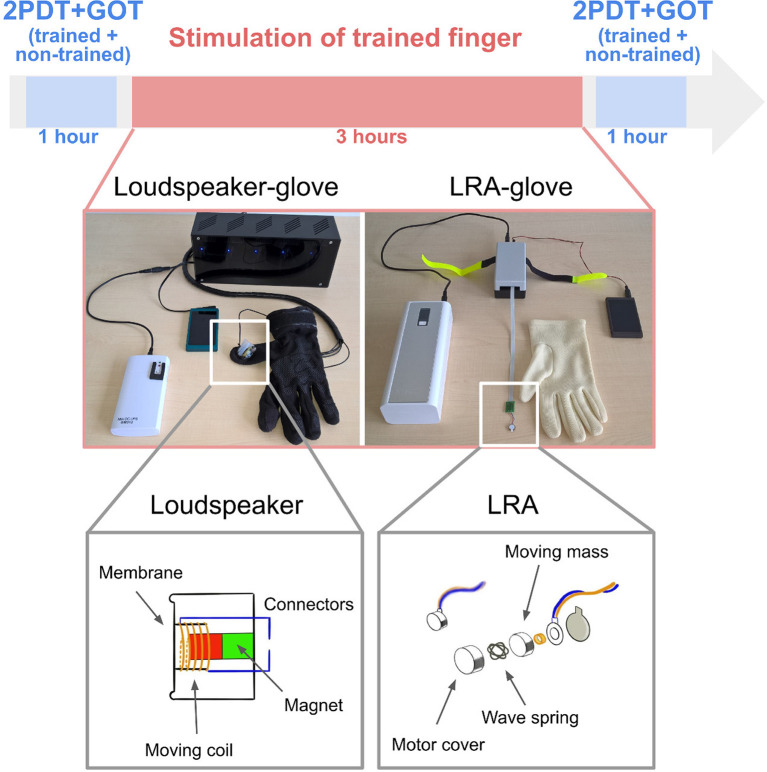
Experimental design and glove systems. Participants were first tested in the two-point discrimination task (2PDT) and the grating orientation task (GOT) both at the index finger and at the middle finger. While wearing the glove, they were then stimulated for 3 h at the trained finger only (index finger for half of the group, middle finger for the other half) using either loudspeaker-based somatosensory stimulation (left) or LRA-based somatosensory stimulation (right). The non-trained finger was covered in the glove, but the stimulation module was off. After the stimulation, participants were tested again in the 2PDT and the GOT both at the trained finger and the non-trained finger.

Two stimulation gloves were developed and tested here for the first time for their effectiveness to induce tactile learning at the hand. The loudspeaker-glove consisted of one mini loudspeaker (8 Ohm) that was mounted inside a commercially available cotton glove using silicone (see Figure 1). The loudspeaker was connected to an Mp3 player via an amplifier. Electricity for the amplifier was provided via a power bank (12 V 2A DC output). Here, only one amplifier was used to stimulate one finger. Four more stimulation modules and four more amplifiers were available for this glove system to potentially stimulate all five fingers at once. If different stimulation protocols for different fingers were needed, different SD cards could be used for different fingers. Powerbank, amplifier, and Mp3 player were placed inside a bag.

The LRA-glove consisted of one LRA (company: Precision Motordrives) attached to the driver, a power bank (50 k mAh, 9–20 V 4A DC output), a controller, and an Mp3 player (see Figure 1). Whereas the loudspeaker-glove, therefore, uses a simple loudspeaker membrane for providing somatosensory stimulation, the LRA-glove uses a magnet attached to a spring that creates vibration via up- and down-movements. Also for the LRA-glove, more than one finger can be stimulated at once if needed, either using the same or a different stimulation protocol via connection to the different actuators. The power bank and the Mp3 layer were placed inside a bag.

### Stimulation Protocol

Both stimulation modules were driven by an audio file that offered a randomized sequence of tactile stimuli according to a previously published protocol (Dinse et al., 2003; Pleger et al., 2003). The stimulation had a duration of 3 h and consisted of a sequence of individual pulses each around 10 ms in length. The average stimulation frequency was 1 Hz, the minimal temporal gap between two successive pulses was 100 ms, and the maximal gap was 3,000 ms.

### Procedure

The experimental procedure is summarized in Figure 1. Before and after the training, participants were tested in the GOT and the 2PDT both at the index finger and at the ring finger. The 2PDT was always tested first. The automated apparatus that was used to test for individual 2PD-thresholds was composed of a disk on which participants placed their hands. The tested finger was fixated onto the disk using adhesive tape. Below the disk there was a rotating wheel, driven by a Piezo motor, that moved the wheel to select one of nine possible pin distances (0.7 mm to 2.8 mm in steps of 0.3 mm, and a one-pin stimulation). The motor automatically moved the selected pin 1 mm upwards for 1,000 ms. The motor was controlled via the software Presentation. During the experiment, each pin distance was presented 10 times in a randomized sequence (new randomization for each participant). After the application of one/two pin/s, participants were asked whether they felt one or two pins on their fingertips. They responded via button press with the left hand.

After the 2PDT was completed for both fingers, the GOT was tested at both fingers using JVP domes. JVP domes are small grating surfaces made out of silicone. The narrower the gratings on a dome are, the more difficult it is to estimate their orientation when applied to the skin surface; estimating orientation is easier for wider gratings. Here, we used grating distances of 0.35 mm, 0.5 mm, 0.75 mm, 1.0 mm, 1.25 mm, 1.5 mm, 2.0 mm, and 3.0 mm that cover the full range between easy and more difficult gratings. All domes could be applied either horizontally or vertically to the fingertip. A custom-made applicator was developed that ensured equal pressure for each application trial. During testing, the hand was positioned upright and the investigated finger was fixated at the table via adhesive tape to reduce finger movements. Via a Matlab-controlled program, the experimenter was informed via loudspeakers which of the 8 possible domes to apply, and in which orientation. Using a random sequence (different for each participant), either horizontal or vertical domes were selected and presented to the fingertip for 1,000 ms. Each dome was presented 14 times (seven times horizontally and seven times vertically). The participant responded via button-press of a computer mouse (left for vertical, right for horizontal).

After the GOT was completed for both fingers, repetitive somatosensory stimulation was applied either via the loudspeaker-glove (*n* = 16 participants, *n* = 8 females) or via the LRA-glove (*n* = 15 participants, *n* = 8 females), and either to the index finger (*n* = 8 for each group, *n* = 4 females) or to the ring finger (*n* = 8 for each group, *n* = 4 females) for the duration of 3 h (see Figure 1). The finger that was stimulated is referred to as the “trained finger” in the following, whereas the finger that was inside the glove but was not stimulated is referred to as the “non-trained finger” in the following. After the stimulation, both fingers were tested again in the GOT and 2PDT using the above-outlined procedures. The total session took approximately 5.5 h.

### Questionnaire

After the testing, participants filled out an 18-item questionnaire where subjective assessments of comfort and handiness of the used stimulation glove were assessed. The questionnaire items are summarized in Figure 3.

**Figure 2:**
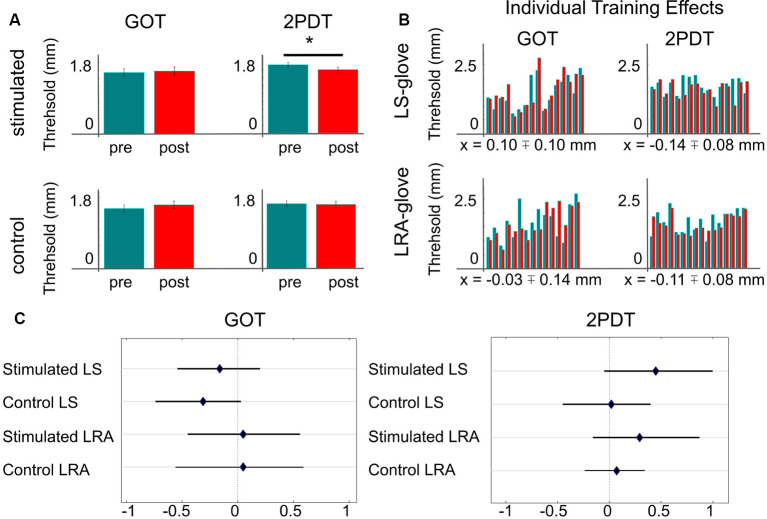
Effect of repetitive somatosensory stimulation using loudspeakers or LRAs on GOT and 2PDT thresholds. **(A)** Averaged thresholds over both stimulators (loudspeaker-glove + LRA-glove). *Indicates significant difference at *p* < 0.05, uncorrected. **(B)** Individual training effects, one bar-pair shows values of one individual; green = pre-training thresholds of the trained finger, red = post-training thresholds of the trained finger, x = mean training effect ± SE; note that negative values indicate better performance after the training compared to before the training, LS, loudspeaker; LRA, linear resonant actuator. **(C)** Effect sizes of stimulation/no stimulation on 2PDT and GOT thresholds, dependent on glove type (LS/LRA); shown are Hedges’ *g* and 95% confidence intervals.

**Figure 3:**
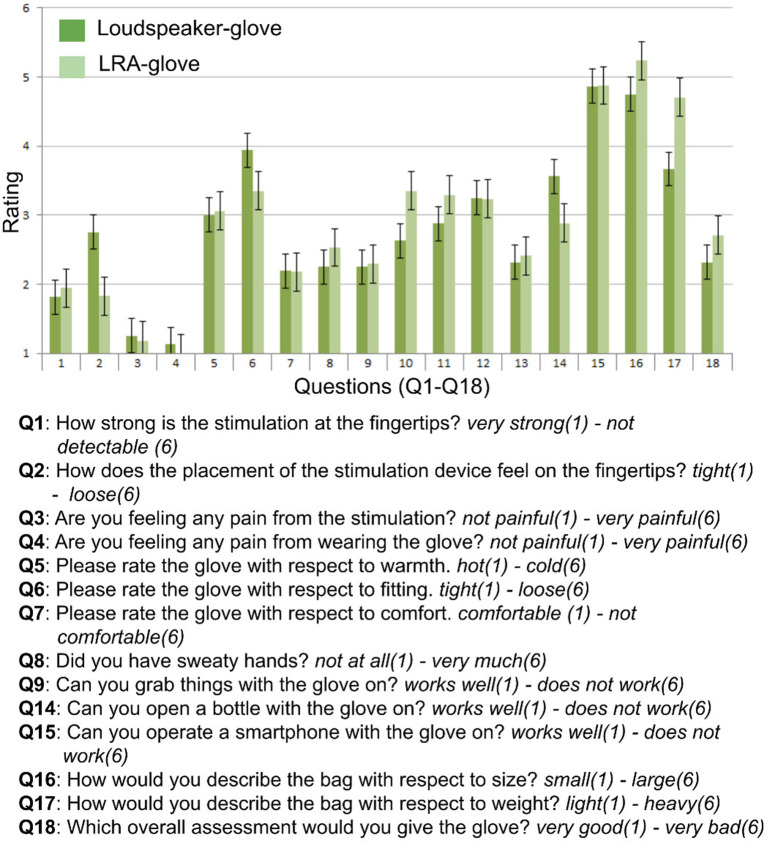
Questionnaire items and results. Rating scales ranged between 1 and 6, questions (Q) and answer dimensions (written in *italics*) are summarized.

### Statistical Analyses

To estimate the 2PDT, psychometric functions were generated via the Statistics and Machine Learning toolbox as implemented in Matlab. Data were fitted via the glmFit function, which offers an iterative weighted least square (IWLS) algorithm to receive maximum-likelihood estimators. IWLS is a standard procedure to fit generalized linear models (Charnes et al., 1976). We used a binary logistic regression to fit the data, i.e., “two” were fitted as percentages across ascending pin distances. The individual two-point discrimination thresholds were taken from the pin distance where the 50% level crossed the fitted sigmoid curve.

To estimate performance in the GOT, the individual 75% discrimination threshold for choosing the correct grating orientation (i.e., vertical vs. horizontal) was estimated using the following calculation:

$$g_{75}=g_{low}+[(0.75-p_{low})/(p_{high}-p_{low})]*(g_{high}-g_{low})$$

where g represents the respective grating and *p* is percentage correct responses. High and low corresponds to the first value that is above or below 75%. In this way, values are interpolated to achieve the grating distance where each participant would reach a 75% threshold.

The Shapiro-Wilk test was used to test for normality of the data using an alpha level of *p* < 0.05. If the hypothesis of a normal distribution was not rejected, we calculated paired-sample *t*-tests to compare the GOT and 2PDT thresholds before and after the training for the trained and non-trained fingers. If the hypothesis was rejected, we used the Mann-Whitney-U-test instead. Effect sizes, confidence intervals and mean differences were computed using the hhentschke/measures-of-effect-size-toolbox as implemented in Matlab^1^ (Hentschke and Stüttgen, 2011). Hedges’ *g* was used to estimate effect sizes (Hedges, 1981). Hedges’ *g* is similar to Cohen’s *d* but outperforms Cohen’s *d* when sample sizes are low. 95% confidence intervals and Hedges’ *g* were computed via bootstrapping 10,000 times. Bootstrapping is a non-parametric statistical test that can be applied both when the data are normal and non-normal distributed. Bootstrapping is particularly suitable for data with small sample sizes. Forest plots were used to visualize effect sizes^2^. A forest plot is a graphical display that illustrates the relative strength of interventions, such as training effects, in different conditions, and is often used in clinical trials to compare the effectiveness of treatments (e.g., Kang et al., 2016). An independent sample *t*-test was used to compare the training effects of the GOT and 2PDT between the loudspeaker-glove and the LRA-glove. Two-tailed, uncorrected *p*-values are reported for the conducted analyses.

## Results

For the 2PDT, all data were normally distributed (*p* > 0.1), except for the data of the non-trained finger after the training, where there was a trend towards the rejection of a normal distribution (*p* = 0.073). We found a significant effect of training in the 2PDT in the trained finger (*t*_(30)_ = 2.07, *p* < 0.05), but not in the non-trained finger (*t*_(30)_ = 0.32, *p* > 0.7, see Figure 2A). The effect sizes of the trained/non-trained fingers were *g* = 0.37/0.04 with bootstrapped 95% confidence intervals ranging from 0.03/−0.22 to 0.77/0.27 (mean difference = 0.12/0.02 mm). For the loudspeaker-glove, the mean training effect was 0.14 mm ± 0.08 mm (SE), and −0.11 mm ± 0.08 mm for the LRA-glove. Note that negative values indicate better performance. Effect sizes for all single comparisons are summarized in Figure 2C. The training effects in the 2PDT for the loudspeaker-glove and the LRA-glove did not differ significantly (*t*_(30)_ = −0.24, *p* = 0.81). There were no significant differences in the 2PDT before the training between the two groups, neither for the trained nor for the non-trained finger (both *p* > 0.7).

For the GOT, all data were normally distributed (*p* > 0.1), except for the data of the non-trained finger before the training (*p* < 0.05). We did not find any statistically significant effect of training in the GOT in the trained or non-trained finger (both *p* > 0.2). The effect sizes of the trained/not-trained fingers were *g* = −0.06/−0.17 with bootstrapped 95% confidence intervals ranging from −0.37/−0.50 to 0.23/0.13 (mean difference = −0.03/−0.09 mm). Effect sizes for all single comparisons are summarized in Figure 2C. The training effects in the GOT between the loudspeaker-glove and the LRA-glove did not differ significantly (*t*_(29)_ = 0.71, *p* > 0.4). There were no significant differences in the GOT before the training between the two groups, neither for the trained nor for the non-trained finger (both *p* > 0.2).

Inspecting individual participant results revealed that for the loudspeaker-glove, 6/16 participants (37.5%) showed an improvement for the GOT, and 10/16 participants (62.5%) showed an improvement for the 2PDT. For the LRA-glove, 10/15 participants (66.6%) showed an improvement for the GOT, and 13/16 participants (81.2%) showed an improvement for the 2PDT (see Figure 2B).

The quantitative inspection of the questionnaire data revealed that the stimulation of both gloves was perceived as strong (Q1) and non-painful (Q3, Q4). Ratings on hotness (Q5), comfort (Q7), sweetness (Q8), and user-friendliness (Q9) were numerically similar between both gloves. Participants also scored numerically similar on the ability to perform grabbing movements (Q13) and to operate the smartphone (Q15) with the glove.

Numerical differences between the loudspeaker-glove and the LRA-glove were observed concerning how the placement of the stimulator felt at the fingertip (Q2) and concerning the fitting of the glove (Q6), where the LRA-glove was perceived as more tight than the loudspeaker-glove. Also, the cables of the loudspeaker-glove were perceived as more user-friendly and less annoying than the cables of the LRA-glove (Q10, Q11). The loudspeaker-glove was also numerically perceived as lighter than the LRA-glove (Q17). Note that the interpretation of the questionnaire data has to be done with caution due to the high number of items and the low number of subjects. Their evaluation should be regarded as qualitative and as an inspiration for future improvements of the glove systems.

## Discussion

Here, we investigated the effectiveness and usability of two mechanical stimulation gloves on somatosensory spatial accuracy via a Hebbian-based learning protocol. We mounted small LRAs into a commercially available cotton glove to apply mechanical stimulation to the fingertips. We compared this system to previously used mechanical stimulators, i.e., loudspeakers, that we also mounted into a cotton glove. The effectiveness of both glove systems to improve tactile spatial discrimination thresholds in two standard tests was explored in a small cohort of young, healthy individuals. The results show no significant effect of somatosensory stimulation on GOT thresholds, but a significant decrease in the 2PDT thresholds after compared to before the training at the stimulated finger only. The loudspeaker-glove improved performance in the 2PDT in 10/16 participants (mean training effect across participants: 0.14 mm), whereas the LRA-glove, improved performance in the 2PDT in 13/16 participants (mean training effect across participants: 0.11 mm). Stimulation gloves may be used in future larger-scale cohort studies on sensorimotor plasticity, rehabilitation, and learning.

Prior studies that used electrical or mechanical stimulation to induce Hebbian learning at the fingertip sometimes reported two or even three times higher learning effects in the 2PDT threshold than those reported here (e.g., 0.2 mm: Dinse et al., 2003; 0.25 mm: Ragert et al., 2008; 0.3 mm: Höffken et al., 2007). However, our lab previously reported a learning effect of 0.11 mm for an experimental setup where repetitive somatosensory stimulation was applied via a loudspeaker membrane that was fixated at the fingertip via adhesive tape (Kuehn et al., 2017). Differences in stimulation protocols or 2PDT task designs may explain this variability. Kattenstroth et al. (2018) reported a learning effect induced by glove-based electrical stimulation of 1.3 mm in the GOT, whereas the 2PDT was not assessed. The learning effect we find here on 2PDT-thresholds is therefore within the lower range of previously reported effect sizes, but still within the expected effect range.

We find an effect of somatosensory stimulation on 2PDT-thresholds but not on GOT-thresholds. It has been shown before that measures of tactile acuity that use the 2PDT and the GOT do not correlate and measure different aspects of tactile spatial acuity (Bruns et al., 2014). For the underlying biological mechanisms, tactile spatial resolution is mediated by the innervation density of slowly adapting Merkel (SAI) afferents (Johnson, 2001), whereas peripheral factors such as skin conformance have rather marginal effects (Peters et al., 2009; Gibson and Craig, 2006; but see Vega-Bermudez and Johnson, 2004). However, tactile spatial acuity depends to a significant extent on central processing as has been evidenced by improvement-related changes in the primary somatosensory cortex as a consequence of tactile learning (Pleger et al., 2003), or as a consequence of visual body perception (Cardini et al., 2011, 2012). These cortex-dependent aspects of tactile spatial perception may differ between the 2PDT and the GOT. As has been argued previously (Bruns et al., 2014), distance-dependent nonlinear lateral interaction processes in the visual cortex have been detected for the presentation of one or more dots in varying distances (Jancke et al., 1999), and similar observations were obtained in the somatosensory cortex for tactile stimulation (Dinse and Jancke, 2001). By contrast, visual presentation of an oriented grating evokes a complex representation that varies across the visual field (Rovamo et al., 1982), and anisotropies have been reported for tactile gratings (Essock et al., 1997; Vega-Bermudez and Johnson, 2004; Gibson and Craig, 2005). 2PDT- and GOT-based effects of improved tactile spatial acuity may, therefore, depend on the size of the stimulated area (Ragert et al., 2008), the intensity of the stimulation, and local vs. global learning effects.

We did not find a significant difference between the learning effects as induced by the loudspeaker-glove compared to the LRA-glove. Effect sizes were marginally higher for the loudspeaker-glove compared to the LRA-glove, whereas numerically more people benefitted from the LRA-glove compared to the loudspeaker-glove. Due to the higher robustness of LRAs compared to loudspeakers, and due to the higher safety of LRAs compared to electrical stimulators, LRA-gloves may be preferred for future clinical applications. Provided the variety of LRAs that are commercially available, future research may determine the optimal system for signal stability, learning efficiency, and stimulator size. It is also worth noting that whereas we here applied a between-subject design, where the factor of the glove (loudspeaker, LRA) was modeled as between-subject factor, in an ideal design, both glove systems would be compared within the same individual (i.e., within-subject design).

Similarly, as in previous studies (Kattenstroth et al., 2018), glove-based somatosensory stimulation was well-tolerated by the participants, and no negative side effects were reported. Sweating or the weight of the battery and drivers were not perceived as severely disruptive by the participants, and the sensation was perceived as non-painful. An advantage of the here introduced glove systems is the possibility to control the stimulation via a smartphone, which would allow easy switching between different stimulation protocols, and intensities. A hardware component that may need modification is the size of the stimulator. The stimulation area is at present confined to a small area of skin, even though the stimulation of larger areas of skin has potentially beneficial effects (Ragert et al., 2008). Furthermore, decreasing the weight of the power banks would significantly increase the comfort to wear the glove in everyday life.

## Data Availability Statement

The datasets generated for this study are available on request to the corresponding author.

## Ethics Statement

The studies involving human participants were reviewed and approved by Ethik-Kommission der Otto-von-Guericke Universität an der Medizinischen Fakultät und am Universitätsklinikum Magdeburg A.ö.R. The patients/participants provided their written informed consent to participate in this study.

## Author Contributions

FT and EK contributed to the conception and design of the study and performed the statistical analysis. FT organized the database and collected the data. EK wrote the manuscript. All authors contributed to manuscript revision, read and approved the submitted version.

## Conflict of Interest

The authors declare that the research was conducted in the absence of any commercial or financial relationships that could be construed as a potential conflict of interest.

## Footnotes

^1^https://www.github.com/hhentschke/measures-of-effect-size-toolbox

^2^https://www.mathworks.com/matlabcentral/fileexchange/71020-forest-plot-for-visualization-of-multiple-odds-ratios
